# How disease extent can be included in the endoscopic activity index of ulcerative colitis: the panMayo score, a promising scoring system

**DOI:** 10.1186/s12876-017-0725-3

**Published:** 2018-01-08

**Authors:** Anita Bálint, Klaudia Farkas, Zoltán Szepes, Ferenc Nagy, Mónika Szűcs, László Tiszlavicz, Renáta Bor, Ágnes Milassin, Mariann Rutka, Anna Fábián, Tamás Molnár

**Affiliations:** 1 First Department of Medicine, University of Szeged, H6720, Korányi fasor 8-10, Szeged, 6720 Hungary; 2 Department of Medical Physics and Informatics, University of Szeged, Korányi fasor 9, Szeged, 6720 Hungary; 3 Department of Pathology, University of Szeged, Állomás u. 2, Szeged, 6725 Hungary

**Keywords:** Ulcerative colitis, Endoscopic Mayo subscore, UCEIS, Endoscopy, Colonoscopy

## Abstract

**Background:**

Colonoscopy plays crucial role in the establishment of the diagnosis, management and follow-up of ulcerative colitis (UC). None of the currently widely used endoscopic scores consider disease extent, and therefore do not correlate with the real severity of UC. Our *aim* was to assess the accuracy of a new score, the Pancolonic Modified Mayo Score that can reflect not only the severity, but the extent of active UC.

**Methods:**

One hundred and four UC patients were enrolled in this prospective study. The Endoscopic Mayo Scores of the involved area of the five colorectal segments were added; furthermore, the sum was multiplied by 3 in case of eMayo ≥2 (range 0 [normal] to 45 [most severe]) to obtain the Pancolonic Modified Mayo Score (panMayo) in order to clearly distinguish the active and inactive disease. We analysed the correlation of panMayo Score with eMayo and Ulcerative Colitis Endoscopic Index of Severity (UCEIS) and complicated disease outcome. We compared the endoscopic indices with serum and faecal inflammatory parameters and Riley Score.

**Results:**

The panMayo Score correlated with eMayo and UCEIS. Every endoscopic score showed correlation with Riley Score, CRP, haemoglobin, haematocrit, serum iron, faecal MMP-9 and calprotectin and also predicted a complicated disease outcome. Only panMayo score correlated exclusively with the extent of UC.

**Conclusions:**

We suggest that this new score gives additional information about disease extent besides disease activity with a strong correlation with laboratory parameters of inflammation and with the other widely used endoscopic indices.

## Background

Ulcerative colitis (UC) is a chronic relapsing inflammatory disease, therefore monitoring disease activity has a great importance in optimising the therapy based on relevant signs and symptoms. Several different scoring systems are available to assess endoscopic disease activity in UC; however, multiple existing indices indicate the lack of an optimal one. On the other hand, mucosal healing becomes one of the desired therapeutic endpoints of inflammatory bowel diseases nowadays [1]. Thus colonoscopy plays a crucial role not only in the establishment of diagnosis, but also in the management and therapeutic decision-making of UC. An exact scoring system for the evaluation of the mucosa is absolutely mandatory for the comparable, standard care of patients with UC all over the world [2].

Truelove and Witts were the first who described a scoring system to evaluate mucosal appearance as a measure of activity for UC [3]. Several scoring indices have evolved during the last decades. The most widely used ones are the Mayo Score, and the validated [4] Ulcerative Colitis Endoscopic Index of Severity (UCEIS) [1]. Both of these scores assess vascular pattern, presence of erythema, friability, erosions, ulcerations and bleeding [5, 6]. Some research confirmed that mucosal healing is associated with better disease outcome [7]. Histologic assessment and scoring is therefore important for the evaluation of UC activity predicting remission rates and risk of malignancy or surgery. Currently, one of the most frequently used histopathological scores is Riley Score [8]. Riley Score incorporates the following histological features: (a) presence of acute inflammatory cell infiltrate (neutrophils in the lamina propria), (b) crypt abscesses, (c) mucin depletion, (d) surface epithelial integrity, (e) chronic inflammatory cell infiltrate (round cells in the lamina propria), and (f) crypt architectural irregularities [7, 8].

Based on a study from Japan, colonoscopy is preferred over sigmoideoscopy for the evaluation of inflammation in UC [9]. Disease extention has a significant implication for prognosis due to the association between extensive UC and colectomy and colorectal cancer; nevertheless, disease localisation and extent determine treatment choice, as well [10]. However, none of the currently widely used endoscopic scores consider disease extent, and therefore they do not correlate with the real severity of UC outcome. Our aim was to assess the accuracy of a new score (Pancolonic Modified Mayo Score) that as a matter of fact is a modified Endoscopic Mayo Subscore (eMayo) in order to reflect not only the activity, but the extent of UC.

## Methods

### Study population

This single-centre, prospective study was carried out on consecutive patients with confirmed diagnosis of UC between January 2011 and October 2015. Diagnosis was based on the European evidence-based Consensus on the diagnosis and management of UC (ECCO) [11]. Disease phenotype and extension were determined according to the Montreal Classification [12] and based on the affected anatomical segments (rectum, sigmoid colon, descending, transverse and cecum/ascending colon). Subjects with UC of varying disease activities and extent were enrolled. Patients with Crohn’s disease or cases with incomplete colonoscopy were excluded. One hundred and four patients were included finally, who were examined by complete colonoscopy which performed by one of three experienced endoscopists (T.M., Z.SZ. and F.N.) in accordance with protocols. All patients had given their written informed consent to the procedure. Observers, who carried out the colonoscopy, rated mucosal lesions (vascular pattern, granularity, friability, ulceration) and the severity of inflammation for each segment and the maximum extension of involvement. Thus, panMayo Score, eMayo and UCEIS were determined in every patient. Biopsy specimens were taken from the most severely affected area for histological examination and scoring. Riley Score was determined for each patient by an experienced gastropathologist.

### Laboratory and faecal markers

We collected blood and faecal samples from every patient to determine specific laboratory parameters for inflammation, namely C-reactive protein (CRP), white blood cell count (WBC), faecal calprotectin and matrix-metalloproteinase (MMP)-9, in addition to haemoglobin, haematocrit, thrombocytes and serum iron level*.* Faecal MMP-9 concentrations were measured using ELISA (Quantikine MMP9 assay, R&D System, UK). One gram of the sample was diluted, homogenised and centrifuged twice; the final supernatant was filtered and stored at −20 °C until analysis that was in accordance with the manufacturer’s instructions [13]. Faecal specimens were stored at −20 °C, thawed and prepared for a calprotectin quantitative lateral flow assay as described by the manufacturer (Quantum Blue, Bühlmann Labortories, Switzerland).

### Pancolonic modified Mayo score

Pancolonic Modified Mayo Score (panMayo) was calculated with the combination of disease extension and severity (Table 1). The eMayo Score of the five colorectal segments (ascendending, transverse, descendending, sigmoid colon and rectum) was determined separately and added afterwards. Finally, the sum was multiplied by the Inflammatory Constant if eMayo was ≥2 at least in one segment to clearly distinguish between the active and inactive disease. Inflammatory constant (IC = 3) was defined at the beginning of the study as the smallest number that can equilibrate the difference between the sum of the inactive and active cases resulting a broad range from 0 [normal] to 45 [most severe, pancolitis with eMayo 3 in each segment]). Inclusion of IC is important, because calculation without IC results the same points in some cases, thus it would be impossible to distinguish different extent and activity by panMayo Score. The most important unmet need for widely used scoring systems in clinical practice that the score simultaneously reflect on the severity and the extension of the disease. The aim of panMayo score was to compensate this gap. If you heard that the eMayo score is 3 points, you do not know that what is the extension of disease and what is therefore exactly the clinical severity of the disease. In contrary, panMayo Score includes both parameters, for example panMayo 45 points is a pancolitis with severe inflammation in all segments.

**Table 1:** Calculation of panMayo score

	Involvement (points)					
eMayo (points)		1	2	3	4	5
	0	0	0	0	0	0
	1	1	2	3	4	5
	2	2 × 3 = 6	4 × 3 = 12	6 × 3 = 18	8 × 3 = 24	10 × 3 = 30
	3	3 × 3 = 9	6 × 3 = 18	9 × 3 = 27	12 × 3 = 36	15 × 3 = 45

Explanation:

Involvement: rectum: 1 point, rectum-sigmoid: 2 points, descending colon: 3 points, transverse colon: 4 points, cecum/ascending colon: 5 points

If no Inflammation (eMayo 0–1 points): eMayo points x involvement [1–5]

If active inflammation is presented in any segment: eMayo points x involvement [1–5] × 3

We analysed the correlation of panMayo Score with the endoscopic indices and the complicated disease outcome. The panMayo Score was compared with Endoscopic Mayo Score and UCEIS to assess the utility of our new score. Complicated disease outcome was defined as the need of hospitalization and/or colectomy during the follow-up.

### Statistical analysis

Correlation of the panMayo Score with clinical, endoscopic and histological indices (partial Mayo Subscore, eMayo Subscore, UCEIS, Riley Score), laboratory measures of disease activity and anaemia (C-reactive protein, faecal calprotectin, faecal MMP-9, serum iron, haemoglobin, haematocrit etc.) were estimated using the multiple correlation analysis with Spearman method. Logrank test was used to determine connection between assessed scores and hospitalisation/colectomy rates. Receiver operating characteristic (ROC) curve analysis was performed to determine the predictive power of the panMayo Score level. Area under ROC curve (AUC), confidence interval for AUC, sensitivity and specificity were calculated. The cut-off level was determined by the maximum value of Youden’s index (sensitivity + specificity-1). The statistical analysis was performed using R (version 3.2.0) statistical software. *P* < 0.05 was considered as statistically significant.

## Results

### Patient characteristics

Out of 104 outpatients, 58 were female and 46 were male; the mean age at enrolment was 43.9 ± 14.7 years. The mean duration of the disease was 8.3 ± 0.7 years. Left-sided colitis was the most common extent (Table 2). On the basis of Mayo Score, 18 patients were in remission, 33 showed mild, 30 moderate and 8 severe disease activity. Nine patients did not require any treatment; however, 65 patients were on 5-ASA, 39 on corticosteroid, 37 on thiopurine, 15 on infliximab and one on cyclosporine-A therapy. Thirty-nine patients were also treated with topically administered drugs. Mean CRP was 21.7 ± 41.4 mg/l, and faecal calprotectin and MMP-9 were also elevated (632.8 ± 874.8 μg/g; 6.8 ± 8.3 ng/l) in the enrolled patients. Strong correlation was shown between the inflammatory markers and the assessed endoscopic scores (see below). Serum and faecal parameters of enrolled patients are presented in Table 3. The mean duration of patient follow-up was 20.7 months (range, 0.3–54.5 months). During the follow-up period, 21 patients required hospitalisation due to relapse (mean: 0.7 times; range 0–15 times) and in 18 cases because of other causes not related to UC (mean: 0.2 times; range: 0–3 times). Nine of 104 subjects underwent colectomy during this period.

**Table 2:** Characteristics of enrolled patients

No. of enrolled patients	104
Male/female	46/58
Mean age (±SD, years)	43.9 (±14.7)
Mean age at diagnosis(±SD, years)	35.3 (±14.7)
Mean disease duration (±SD, years)	8.3 ± 0.7
Localisation of disease:	
Rectum	23
Sigmoid colon	28
Descending colon	18
Transverse colon	12
Ascending colon (±cecum)	23
Disease activity	
The mean of eMayo Score (mean ± SD, points)	1.8 ± 1.2
The mean of UCEIS (mean ± SD, points)	6.3 ± 2.4
The mean of panMayo Score (mean ± SD, points)	14.2 ± 13.7
Therapy:	
5-ASA	65
Corticosteroids	39
Thiopurines	37
Cyclosporine-A	1
Biological therapy: infliximab	15
Topical	39
No treatment	9

**Table 3:** Laboratory markers of enrolled patients

Laboratory parameter	value
CRP (mean ± SD, mg/l)	21.7 ± 41.4
WBC (mean ± SD, G/l)	7.8 ± 3.3
Thrombocytes (mean ± SD, G/l)	299.3 ± 99.9
Haematocrit (mean ± SD, %)	39.1 ± 5.1
Haemoglobin (mean ± SD, g/l)	130.6 ± 19.7
Serum iron (mean ± SD, umol/l)	12.3 ± 6.2
Faecal calprotectin (mean ± SD, μg/g)	632.8 ± 874.8
Faecal MMP-9 (mean ± SD, ng/ml	6.8 ± 8.3

**Table 4:** Correlation between assessed endoscopic scores and clinical/laboratory markers

	eMayo		UCEIS		panMayo	
	Spearman coefficient (R)	p	Spearman coefficient (R)	p	Spearman coefficient (R)	p
Disease extension	−0,04	0,687	−0,078	0,429	0,339	<0.001
partial Mayo Score	0,714	<0.001	0,714	<0.001	0,692	<0.001
CRP	0,306	0,004	0,481	<0.001	0,481	<0.001
Leukocytes	0,283	0,007	0,276	0,009	0,278	0,009
Haematocrit	−0,237	0,025	−0,3	0,004	−0,3	0,004
Haemoglobin	−0,238	0,024	−0,337	0,001	−0,337	0,001
Thrombocytes	0,175	0,102	0,307	0,003	0,307	0,003
Serum Iron	−0,352	0,001	−0,499	<0.001	−0,499	<0.001
Riley Score	0,724	<0.001	0,691	<0.001	0,691	<0.001
Calprotectin	0,399	0,019	0,349	0,043	0,349	0,043
MMP-9	0,505	<0.001	0,554	<0.001	0,554	<0.001

### Comparison between indices

#### PanMayo score

The mean panMayo Score was 14.2 ± 13.7 points (range, 0–45 points). Inactive and active disease was demonstrated in 45 and 59 patients. The ROC analysis revealed an AUC of 0.884 (*p* < 0.001, 95% CI: 0,822–0,946). The sensitivity and specificity of panMayo in the establishment of disease activity was 100% and 69.2%.

**Fig. 1:**
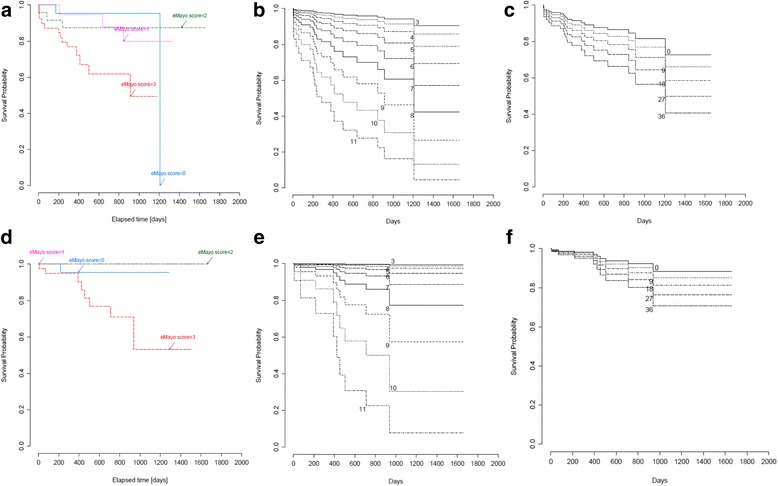
Logrank analysis of hopitalisation free survival shown for: **a** eMayo Score, **b** UCEIS, **c** panMayo Score, and of colectomy free survival shown for: **d** eMayo Score, **e** UCEIS, **f** panMayo Score

This new score correlated significantly with disease extent (*R* = 0.3; p < 0.001) and partial Mayo Score (*R* = 0.7; p < 0.001). In addition, it showed a strong association with the histological assessment, namely Riley Score (R = 0.7; p < 0.001). We found panMayo Score to correlate with CRP (*R* = 0.5; p < 0.001), faecal MMP-9 (R = 0.5; p < 0.001), calprotectin (*R* = 0.35; *p* < 0.043), leucocyte (R = 0.3; *p* = 0.009), thrombocyte levels (*R* = 0.3; *p* = 0.003), and also with haemoglobin (*R* = −0.3; *p* = 0.001), haematocrit (R = −0.3; *p* = 0.004) and serum iron level (*R* = −0.5; *p* < 0.001) (Table 4). Significant association was shown between panMayo Score and hospitalisation rate (*p* = 0.043). After reviewing our data, we observed a relationship between panMayo Score as a linear variable and colectomies, although this could not be substantiated by statistical analysis (*p* = 0.6) (Fig. 1).

#### Endoscopic Mayo score

The mean eMayo Score was 1.8 ± 1.2 points (range, 0–3 points) in this study. Inactive and active disease was presented in 41 and 63 patients. eMayo Subscore showed high correlation with partial Mayo Score (*R* = 0.7; *p* < 0.001), Riley Score (R = 0.7; *p* < 0.001), CRP (*R* = 0.3; *p* = 0.004) and faecal MMP-9 (*R* = 0.5; p < 0.001), but also with faecal calprotectin (*R* = 0.4; *p* = 0.019), serum iron (*R* = −0.4; *p* = 0.004) and leucocyte level (*R* = 0.3; *p* = 0.007), haemoglobin (*R* = −0.3; *p* = 0.02) and haematocrit (*R* = −0.2; p = 0.02). We did not find association with disease extent (*R* = −0.04; *p* = 0.7) and thrombocytes (*R* = 0.2; *p* = 0.1) (Table 4). We found that eMayo is strongly connected with hospitalisation (*p* = 0.028) and colectomy rates (*p* = 0.008).

#### UCEIS

The mean UCEIS was 6.3 ± 2.4 points (range, 3–11 points). Inactive and active disease was shown in 41 and 63 patients. UCEIS correlated with partial Mayo Score (R = 0.7; *p* < 0.001), Riley Score (R = 0.7; p < 0.001), faecal MMP-9 (R = 0.5; p < 0.001), faecal calprotectin (*R* = 0.35; *p* = 0.043), CRP (R = 0.5; p < 0.001), leucocyte count (R = 0.3; *p* = 0.009), thrombocytes (R = 0.3; *p* = 0.003), haemoglobin (R = −0.3; *p* = 0.001), haematocrit (R = −0.3; p = 0.004), and serum iron level (*R* = −0.5; p < 0.001), but not with disease extent (R = −0.04; p = 0.7) (Table 4). UCEIS showed a significant connection with hospitalisations (p < 0.001) and colectomies (p < 0.001).

#### Correlation between indices

The panMayo Score correlated with eMayo (*R* = 0.853; p < 0.001) and UCEIS (*R* = 0.783; p < 0.001), and eMayo with UCEIS as well (*R* = 0.932; p < 0.001).

## Discussion

In this study we assessed the diagnostic value of a new, modified endoscopic score. The panMayo Score revealed a significant correlation with laboratory parameters of inflammation and anaemia, disease extension and histological activity of UC. Furthermore, despite the modification it showed 100% sensitivity with the “mother” endoscopic disease activity eMayo Score.

Use of endoscopic scoring systems is recommended for the evaluation of the prognosis and efficacy of treatment. The ideal score would be easy to use, reproducible, reliable, responsive to changes and validated [14]. Several endoscopic scoring systems are available for the assessment of disease severity, although only UCEIS and ulcerative colitis colonoscopic index of severity (UCCIS) [15] received formal validation [16]. UCEIS with a total score of 3 to 11 (or modified: 0–8) points was created based on vascular pattern, bleeding, and erosion/ulceration [17]. The study of Corte et al. showed that UCEIS has a predictive value also, because it was associated with the outcome of acute severe UC [18]. The Mayo Score takes into account four variables; these are stool frequency, rectal bleeding, the physician’s global assessments and findings at endoscopy, namely the eMayo Score [17]. eMayo Score differentiates between endoscopically inactive, mild, moderate and severe disease. Every endoscopic score indicates the severity of inflammation, but not the extent of the inflamed colon which is a critically important detail for the optimisation of the therapy [10, 19]. The panMayo Score describes the bowel mucosa similarly to eMayo, but it also includes disease extent. We found strong correlation between panMayo Score and UCEIS, and eMayo as well. In addition, panMayo showed significant association with serum and faecal inflammatory markers. Therefore, this new score can correctly demonstrate the activity of UC and revealed unique correlation with disease extent hereby indicating one of the significant factors of disease outcome. Patients with initial diagnosis of pancolitis tend to present with more frequent complications, extraintestinal manifestations, occurrence of colorectal cancer is higher among them, and they are more likely to require immunosuppressive or surgical therapy [20, 21].

Despite general principles that treatment decisions should be based on disease activity, pattern of the disease and its distribution, colonoscopy and its evaluation may also play a crucial role in the therapy [19]. The panMayo Score correlates with inflammatory markers, and with parameters of anaemia as well, that is the most common systemic complication or extraintestinal manifestation of UC [22]. Anaemia is a disease activity related condition (triggered by blood loss and inflammation) in UC, moreover sometimes it is the only sign of an ongoing inflammation [23]. Furthermore, in another study, anaemia was shown to be more common in patients with UC requiring immunosuppressive therapy [24]. Nevertheless, anaemia in a population-based IBD cohort was associated with the extent of UC [25] and in our previous study the need for blood transfusion was a significant predictor of a subsequent colectomy [26]. It should be noted that disease location and extension has a great influence on the prognosis: approximately one third of patients with extensive UC will require colectomy during the disease course [27]. Nine of our patients had colectomy during the follow-up period. The panMayo Score as well as eMayo Score, and UCEIS have a predictable value on outcome in our study (Fig. 1).

To improve the accuracy of the endoscopic Mayo score, another work group had also created an alteration on the eMayo Score. The Modified Endoscopic Mayo Score which, similarly to the panMayo Score, considers the distribution of UC, showed good correlation with clinical, biological and histological activity of UC. However, calculation seems to be slightly difficult: after counting Modified Score and Extended Modified Score, the Modified Mayo Endoscopic Score [28] was obtained by dividing the Extended Modified Score with the number of segments with active inflammation.

The limitation of panMayo Score is that it can be calculated after a total colonoscopy which is not recommended in extraordinarily severe cases. The use of IC makes the calculation of panMayo Score slightly complicated, however, it is necessary for the clear differentiation of active from inactive disease. On the other hand, the general problem with every scoring system is that the reproducibility of endoscopic scores remains suboptimal as a study from Italy reported; assessment depends on the expertise of the gastroenterologist [8]. The key of consistent evaluation is suggested to be a standardized system of description and endoscopists’ training [29].

Summarizing our data, panMayo Score is a new, disease extent-related endoscopic score (created with a slight modification of eMayo) for the assessment of disease activity in UC, that showed excellent sensitivity and a good correlation with the parameters of inflammation and anaemia, Riley, eMayo Score and UCEIS. Nevertheless, it can be more favourable than the above mentioned endoscopic scores because panMayo Score gives additional information about disease location besides disease activity with a strong correlation with laboratory parameters of inflammation and anaemia.

## Conclusion

There are many indices available to assess the endoscopic activity of ulcerative colitis, but all of them have some limitations. The panMayo Score provides additional information about the extent of the disease besides the disease activity.

## Abbreviations

CRP: C-reactive protein
eMayo: Endoscopic Mayo Subscore
MMP-9: Matrix-metalloproteinase-9
panMayo: Pancolonic Modified Mayo Score
UC: Ulcerative colitis
UCEIS: Ulcerative Colitis Endoscopic Index of Severity
WBC: White blood cell
